# Molecular taxonomic study of *Strongyloides* spp. in Sri Lanka with emphasis on *Strongyloides fuelleborni* on the premises of a Sri Lankan university

**DOI:** 10.3389/fpara.2026.1821438

**Published:** 2026-07-08

**Authors:** Veroni de Ree, Lakshitha Kumara, W.A.A.H. Kalhari, Sandra S. Gyarteng Mensah, Dorothee Harbecke, H.K.S. de Zoysa, Ajith I. Rathnayake, Jagath C. Ranasinghe, Christian Rödelsperger, Kosala Weerakoon, Adrian Streit

**Affiliations:** 1 Department of Integrative Evolutionary Biology, Max Planck Institute for Biology, Tübingen, Germany; 2 Department of Bioprocess Technology, Faculty of Technology, Rajarata University of Sri Lanka, Mihintale, Sri Lanka; 3 Department for Theoretical and Applied Biology, Kwame Nkrumah University of Science and Technology, Kumasi, Ghana; 4 Sunyani Technical University, Sunyani, Ghana; 5 District General Hospital, Nawalapitiya, Sri Lanka; 6 Department of Parasitology, Faculty of Medicine and Allied Sciences, Rajarata University of Sri Lanka, Saliyapura, Sri Lanka

**Keywords:** molecular taxonomy, Sri Lanka, *Strongyloides fuelleborni*, *Strongyloides stercoralis*, strongyloidiasis, zoonosis

## Abstract

**Introduction:**

*Strongyloides stercoralis* is the main cause of human strongyloidiasis, but increasingly, presumably mostly zoonotic, human infections with *S. fuelleborni* are reported. Recently, a number of studies investigated the population structures, host specificities and zoonotic potentials of these two species, using molecular taxonomy. This is the first such study from Sri Lanka.

**Methods:**

We collected stool samples from humans, dogs and monkeys and used the Baermann after culture method to isolate individual live *Strongyloides* worms and determined parts of the *cox-1* (mitochondrial) and the 18S rDNA (nuclear) and, for a subset, whole genome sequences.

**Results and discussion:**

Despite a predicted high prevalence, we did not detect any *Strongyloides* in 126 human stool samples from the plantation sector. This may reflect the impact of improvements in sanitation and hygiene, reducing the worm burden to below our detection threshold. On the other hand, we observed a rather high incidence of *S. fuelleborni* in monkeys from within a university campus. Unexpectedly, also the two *Strongyloides* positive dogs we found on campus passed *S. fuelleborni*, one along with the expected *S. stercoralis*. Based on the mitochondrial sequences, the *S. fuelleborni* formed three subclusters within the previously described Asian clade. At the nuclear 18S rDNA locus, all *S. fuelleborni* carried haplotypes that had previously been described in Asia but some were heterozygous for one Asian allele and one that had so far been considered Africa specific. This suggests that Sri Lanka might be a hybridization zone for the two clades. The *S. stercoralis* found in this study clustered with the previously proposed ‘human and dog shared’ type of *S. stercoralis*.

**Conclusions:**

The presence of possible hybrids between the African and the Asian clade of *S. fuelleborni* and the presence of this species in dog faeces are interesting findings that need to be confirmed in additional studies. The presence of *S. stercoralis* and *S. fuelleborni* in animal faeces within the University premises, including the dormitory and the swimming pool area, raises concerns of possible exposure of the University community to these parasites in spite of sanitary conditions that render human to human transmission unlikely.

## 1 Introduction

The nematode parasite *Strongyloides stercoralis* is the main causing agent of human strongyloidiasis (Barroso et al., 2019; Nutman, 2017; Rodriguez-Perez et al., 2025). According to recent estimates, the number of infected people worldwide exceeds 600 million (Buonfrate et al., 2020). *S. stercoralis* infections are most common among socio economically disadvantaged people in tropical and subtropical regions (Beknazarova et al., 2016). However, *S. stercoralis* is not restricted to these settings and cases in highly developed countries outside of the tropics are regularly reported (Barroso et al., 2019; Buonfrate et al., 2021). While the majority of these cases involve travellers who were probably infected in tropical or subtropical countries, evidence for local transmission has been reported in, for example, Slovakia (Strkolcova et al., 2017) and Spain (Rodriguez-Perez et al., 2025). In *S. stercoralis*, a fraction of the progeny of the parasitic worms mature to infective third stage larvae while still within the host (Nutman, 2017). This leads to a chronic infection that can last for decades. While the chronic *S. stercoralis* infections frequently remain at a very low worm burden and many infections progress with little or no clinical manifestations, under some circumstances, in particular immuno-deficiency of the carrier, the auto infective cycle can accelerate leading to hyperinfection syndrome and disseminated strongyloidiasis, which are usually fatal if not treated in time (Keiser and Nutman, 2004; Nutman, 2017). While most human *S. stercoralis* infections appear to be acquired from other humans, *S. stercoralis* has been described in animals, notably non-human primates, dogs and cats, and the possibility of zoonotic transmission, in particular from dogs, has been the subject of an ongoing controversial discussion for more than a hundred years (Bradbury and Streit, 2024; Jaleta et al., 2017; Liu et al., 2025; Nagayasu et al., 2017).

It has long been known that a second species of *Strongyloides*, *S. fuelleborni*, which is the predominant *Strongyloides* in old world non-human primates (NHP), is found in humans (Zhao et al., 2025a). Within the genus *Strongyloides*, *S. stercoralis* and *S. fuelleborni* belong to different groups, such that *S. fuelleborni* is more closely related with the ruminant parasite *S. papillosus* than with *S. stercoralis* (Richins et al., 2025; Zhao et al., 2025a). A diagnostically distinguishing feature is that *S. fuelleborni* eggs are found in fresh stool samples while essentially all *S. stercoralis* larvae hatch within the host such that only young larvae are present, even in very fresh stool samples (Ashford, 1989; Viney et al., 1991). While in Africa human *S. fuelleborni* cases were regularly reported and there is evidence for human to human transmission, in Asia human *S. fuelleborni* were considered rare and exclusively zoonotic (Barratt and Sapp, 2020; Zhao et al., 2025a). However, recent studies suggested that also in Asia human *S. fuelleborni* infections might be more common than previously assumed (de Ree et al., 2024; Thanchomnang et al., 2017; Zhao et al., 2025a, Zhao et al., 2025b). In *S. fuelleborni* no auto-infective cycle and consequentially no hyperinfection and dissemination have been reported. This species is therefore of less concern than *S. stercoralis*, but our understanding of the clearly existing clinical and sub-clinical consequences of *S. fuelleborni* infections is very limited (Zhao et al., 2025a). Further, it is a confounding factor in the *S. stercoralis* diagnostics, in particular if diagnostic methods that involve faecal culturing and the detection of *Strongyloides* larvae are used, but also cross reactivity in serological diagnostics cannot be excluded and has to our knowledge not been systematically tested.

Recent molecular taxonomic studies revealed a clear geographic genetic structure within the species *S. fuelleborni* with an African and an Asian clade, irrespective of the host species (Barratt and Sapp, 2020; Ko et al., 2023; de Ree et al., 2024; Richins et al., 2025). In contrast, rather little geographic genetic structure has been found in *S. stercoralis* (see Barratt and Sapp, 2020; de Ree et al., 2024 and references therein) and this species has been proposed to have spread over the world relatively recently (Nagayasu et al., 2017). However, in *S. stercoralis* there is genetic structuring according to host species. The existence of dog specific types along with types that can occur in humans and in dogs was first described by Jaleta et al. (2017) and Nagayasu et al. (2017). Barratt and Sapp (2020) later argued that also the “human and dog” type might consist of sub types only some of which can indeed infect both hosts. While the human and dog type appears to be cosmopolitan, the dog specific type has so far been found only in Southeast Asia, Australia and Bangladesh (Jaleta et al., 2017; Nagayasu et al., 2017; Beknazarova et al., 2019; de Ree et al., 2024; Liu et al., 2025).

At the moment, global sampling and molecular taxonomic analysis of both human infective species of *Strongyloides* is very patchy and incomplete. To our knowledge, no molecular taxonomic information for any species of *Strongyloides* has been reported from Sri Lanka.

The overall prevalence of soil transmitted helminth (STH) infections in Sri Lanka has dropped dramatically over the last 100 years (reviewed by Jayakody et al. (2024), compare for example Ediriweera et al (2019), who reported a prevalence of 0.97% for STH in school children in 2017 and Chellappah, 1938, who found a 90.5% prevalence of hookworms in an island-wide survey, conducted in 1924-25). However, a much higher prevalence compared to the overall national prevalence has been steadily reported in the plantation sector over the years (Jayakody et al., 2024). This reference estimated an STH prevalence of 33.4% in plantation communities as opposed to 6.9% in the general (non-plantation, non-slum) community. For the central province, where the study described in this paper was conducted, Ediriweera et al. (2019) reported an STH prevalence of 0.42% overall but 9.02% in the plantation sector.

Mass drug administration (MDA) as a control measure has been employed in Sri Lanka since 1930 (Chellappah, 1938). Later, the MDA efforts have been more directed towards targeted populations such as the plantation sector, where a major deworming effort started in 1994 with a biannual single dose of 500mg mebendazole but was discontinued due to lack of funds in 2005 (Gunawardena et al., 2011). Another deworming program was carried out from 2013 to 2018, including the plantation sector, with the same medication strategy as before for this population (Ediriweera et al., 2019).

The aforementioned surveys were general STH surveys focused on ascariasis, trichuriasis and hookworm infections. Strongyloidiasis in Sri Lanka is not reported in these surveys, which is not surprising as the detection method used in these surveys is mainly Kato-Katz, which mainly detects eggs in the faeces and is likely to miss *S. stercoralis*, which sheds hatched larvae, not eggs (Knopp et al., 2014).

The first strongyloidiasis case in Sri Lanka was reported in 1983 (Wijesundera et al., 1983). Contrary to the trend with STH in general, there has been an increasing number of case reports of strongyloidiasis in the recent past in Sri Lanka (Gunathilaka et al., 2020; Karunatilaka et al., 2021; Morel et al., 2007, Morel et al., 2022; Rodrigo et al., 2012). However, all of these reports, except for (Rodrigo et al., 2012), are about immunocompromised patients. In the context of their global prevalence estimation study (Buonfrate et al., 2020), estimated the strongyloidiasis prevalence for Sri Lanka at >10-15%. A recent cross-sectional study conducted during 2021–23 and including more than 100 immunocompromised patients showed a prevalence of 0.6% for direct smear and culture methods, 11.4% for the PCR-based (amplification of a 129 bp fragment containing *ITS1* and *5.8S rDNA* sequences followed by end point analysis [gel electrophoresis and sequencing]) methods and 16.4% for serological (ELISA, DRG International - EIA 4208) methods, illustrating the difficulties in *S. stercoralis* diagnostics (Weerasekera et al., 2024).

Although, based on the 2017 island-wide survey, the Anuradhapura district was recognised as a low-prevalence area for STH in general, a pilot study in 2023 showed 5% prevalence of *S. stercoralis* in the school children (Jayakody et al., 2024).

The few studies that looked at *Strongyloides* spp. in dogs in Sri Lanka reported prevalences between 11% and 26% (Amarasingha et al., 2024; Bandaranayaka et al., 2019; Perera et al., 2013). We are not aware of any report of *S. fuelleborni* in humans in Sri Lanka. However, given the diagnostic difficulties mentioned above, if might well be that in some instances *S. fuelleborni* was mistaken for *S. stercoralis*.

In Sri Lanka, there are 5 non-human primate species, including 3 monkey species: purple-faced langur (*Trachypithecus vetulus* – endemic with four subspecies; *T.v. vetulus, T.v. nestor, T.v. philbricki, T.v. monticola*), toque macaques (*Macaca sinica* - endemic with 3 subspecies; *M.s. aurifrons, M.s. sinica, M.s. opisthomelas*) and Grey Langurs (only found *Semnopithecus priam thersites;* the endemic subspecies) and 2 loris species: *Loris tardigradus* (endemic with 2 subspecies; *L.t. tardigradus* and *L.t. nycticeboides*) and *Loris lydekkarianus* [with 2 out of 4 subspecies found in the island and are endemic; *L.l. nordicus* and *L.l. grandis* (Nahallage et al., 2008)].

Sepalage and Rajakaruna (2020) found *Strongyloides* in a Grey Langur from the Udawalawe national park. A study conducted in 2018–19 showed 14.3% overall *Strongyloides* spp. prevalence in toque macaques (*M.s. sinica* and *M.s. aurifrons*) (Thilakarathne et al., 2021). This study also included samples from free-roaming monkey troops within the University of Peradeniya. Fernando et al. (2022) sampled faeces from all three toque macaque sub-species from all three different climate zones and three habitats (urban, suburban and wild) and tested them for the presence of gastrointestinal (GI) parasites. In all three habitats, the most common GI parasite was *Strongyloides* spp. with the prevalence ranging from 30% (*M.s. opisthomelas* in the wild habitat) to 85% (*M.s. sinica* in the urban habitat). Interestingly, urban and suburban monkeys had a higher prevalence of *Strongyloides* spp. than the ones from the wild habitat. To our knowledge, so far, no molecular taxonomic investigation from any *Strongyloides* spp. in Sri Lanka has been conducted.

The aim of this study was to contribute molecular taxonomic data on human-infective *Strongyloides* spp. from Sri Lanka to global phylogenetic and molecular taxonomic datasets, rather than to conduct a sensitive prevalence assessment. To this end, we attempted to sample *Strongyloides* from humans, dogs and monkeys and subject them to molecular taxonomic analysis using the Small ribosomal Subunit Hypervariable Region 1 and 4 (*SSU* HVR-I and HVR-IV) as nuclear and a portion of the *cox-1* (encoding cytochrome c oxidase subunit 1) as mitochondrial markers. Because we wanted to correlate mitochondrial and nuclear markers in individual worms, we opted for diagnostic methodology that allows the isolation of live worms, of which currently Baermann after culture is considered the most sensitive (Gelaye et al., 2021).

## 2 Materials and methods

### 2.1 Ethics statement

Both human and animal faecal sample collection and processing were approved by the Ethics Review Committee of the Faculty of Medicine and Allied Sciences, Rajarata University of Sri Lanka (ERC/2023/11). Participants volunteered in the study and gave informed written consent. In the case of children, the guardian of the child gave written consent. Interested participants were given information verbally in a group setting, followed by a one-on-one interaction regarding the study, where a written information sheet was also given, along with the opportunity to ask questions. All the interactions with the potential participants were done in their preferred language (Sinhalese or Tamil). Then the participants were instructed on how to properly collect the sample and handed a sample collection kit. Returning the sample was entirely voluntary.

### 2.2 Sample collection

Since this was a molecular taxonomic study, sample collection was optimised for the isolation of live individual worms and not for diagnostic sensitivity. Human stool samples were collected from all residents of a Tea Estate, in Nuwara Eliya, Sri Lanka, who volunteered to participate in the study. The participants reported no recent history of being treated with anthelmintics. A sample collection kit was handed to the participants. The kit included a diagrammatic instruction in both Sinhalese and Tamil languages on how to collect the samples, a pair of gloves to wear during the collection, a tissue paper to catch the faecal sample, a labelled sample collection jar with a spoon and a screw-top lid, a piece of news paper to cover the jar with faeces for privacy, and a Ziplock bag to safely bring back the jar. The next day, the sample jars were collected. A total of 200 sample collection kits were distributed and 125 samples were returned, of which 117 were sufficiently large to be analysed with our methodology. Five freshly deposited samples from free-roaming dogs in the neighbourhood were also collected. Each sample was mixed on site with approximately equal volumes of charcoal to facilitate air exchange, and water was added as necessary to moisturise the sample well while not making it watery. The samples were then transported to the Faculty of Technology of Rajarata University of Sri Lanka on the same day of collection, for subsequent processing and analysis. During the entire process, the samples were kept at ambient temperature. In addition, 9 samples collected from school children in Rambewa, Anuradhapura, were also investigated.

On the premises of the Rajarata University of Sri Lanka, numerous monkeys and dogs roam freely. They live in close proximity to the student dormitories and sports facilities (e.g. the swimming pool). Freshly deposited faecal samples from monkeys (29) and dogs (8) were collected. These samples were treated as described above. Sample collection was done in June 2024.

### 2.3 Sample processing

All the samples were processed at the Faculty of Technology of the Rajarata University of Sri Lanka, where they were incubated for 48 hours and remoisturized on a need-to basis. Samples were analysed using the Baermann technique and the sediment was observed under a stereo dissecting microscope for the presence of worms as described (Zhou et al., 2019b). Worms were then individually preserved in 80% ethanol and brought back to the Max Planck Institute for Biology, Tübingen for further analysis.

### 2.4 Single worm lysis and *SSU* and *cox-1* genotyping

Single-worm lysis was done as described (Zhou et al., 2019b) and the *SSU* HVR-I and *cox*-1 genotyping was done using the primers listed in (Zhou et al., 2019a, Zhou et al., 2019b). For the *SSU* the HVR-I primers RH5401 and RH5402 were used and for *cox*-1, the primers ZS6985 and ZS6986 were used. Although the primers used for *cox*-1 genotyping were designed for *S. stercoralis*, they successfully amplified the *S. fuelleborni* sequence in de Ree et al. (2024). Sequencing of the PCR products and sequence analysis were also done as described in de Ree et al. (2024). New sequences were submitted to GenBank, accession numbers PX993795-PX993809. The information for each worm is given in **Supplementary Table 1**.

### 2.5 Whole genome sequencing and extraction of the *cox-1* and *SSU* HVR-I and HVR-IV sequences

Library preparation for 150bp paired-end Illumina sequencing was done as described (Beiromvand et al., 2024), except that 12-18µl of lysate were used as starting material instead of 10µl, and the bead cleanup after the pooling was skipped. For 30 single worms (of 35 attempted), the library preparation was successful, and 2.2 nM from each sample was pooled and submitted for sequencing to the in-house sequencing facility of the Max Planck Institute for Biology, Tübingen, which uses an Illumina NexSeq 2000 instrument. All whole genome sequencing data sets were submitted to the European Nucleotide Archive under the study accession PRJEB106561.

#### 2.5.1 *cox*-1 tree

Sequences from whole genome sequencing were aligned to the *S. fuelleborni* whole mitochondrial sequence arrangement A (OL505577.1 , Ko et al., 2023). Read alignment was done using the bioinformatic pipelines described in (Beiromvand et al., 2024), and the resulting SAM files were converted to BAM files. Then the alignments were loaded to IGV and the consensus sequences were extracted and saved as.dna-files as described in (de Ree et al., 2024), which were then loaded onto SnapGene software (from Dotmatrics; available at snapgene.com) and the same 552bp of *cox*-1 sequences as used in our previous studies (Beiromvand et al., 2024; de Ree et al., 2024) were extracted.

The *cox*-1 sequences (either derived from PCR and Sanger sequencing or extracted from WGS) were used to generate Neighbour Joining (NJ) trees with 1000 bootstrap values, using MEGA12 (Kumar et al., 2024) after aligning them with the MUSCLE function in the software.

#### 2.5.2 *SSU* hypervariable regions (HVR-I and HVR-IV)

GenBank entry AB272235.1 was used as the reference in this analysis. Read alignment and consensus sequence extraction were done as described above for *cox*-1 sequences. Sequences were checked manually using IGV and SnapGene software (from Dotmatrics; available at snapgene.com) to determine the haplotypes for HVR-I and HVR-IV (see **Supplementary Table 1**).

### 2.6 Population genomic analysis

The whole genome sequencing reads were aligned with the BWA (version0.7.17-r1188 with mem program) software to the closest available reference genome which was the one of *S. papillosus* (version WBPS19 from WormBaseParaSite) (Hunt et al., 2016; Li and Durbin, 2009). For comparison, we included the whole genome sequencing data for an *S. papillosus* strain (Europeannucleotide archive accession SRR8858637). Variant calling was done with the programs samtools and bcftools as described previously (de Ree et al., 2024; Li et al., 2009). The total number of single nucleotide variants (SNVs) and the number and fraction of heterozygous SNVs were determined for each sample. In order to reconstruct a genome-wide phylogeny, we concatenated homozygous variants that were genotyped in all samples and computed a neighbour-joining tree from the resulting alignment (de Ree et al., 2024).

## 3 Results and discussion

### 3.1 Animal and human stool samples from Nuwara Eliya and Anuradhapura

Using our detection method (faecal culture followed by Baermann funnel - see Materials and methods) we did not detect parasitic nematodes in any of the 126 human samples (117 from Nuwara Eliya, 9 from Anuradhapura). In three samples from Nuwara Eliya, we found worms that we believe to be free-living. Considering the sequence of the 431bp *SSU* HVR-I fragment, we found worms that were 100% identical with GenBank entry EU196004 (*Auanema rhodensis*, samples SL079 and SL038), worms whose best BLAST hit (84% identity) was GenBank entry OR632674 (*Tokorhabditis artipennis*, samples SL019 and SL038) and worms whose best BLAST hit (84% identity) was MG669838 (*Litoditis* spp., sample SL038). All these taxa are free-living worms (Kanzaki et al., 2017) and we assume that they were contaminants that arrived in the stool sample after deposition. We had noticed *Tokorhabdhitis* spp. in human samples before in Bangladesh (de Ree et al., 2024). *Tokorhabdhitis artipennis* has been found on dung beetles (Ragsdale et al., 2022). Dung beetles can colonise faeces very quickly, sometimes within seconds (Mohr, 1943).

In 4 out of 5 dog samples collected from Nuwara Eliya we found live worms other than *Strongyloides*. Molecular analysis, based on the same 431bp *SSU* HVR-I fragment as above, confirmed that 3 of the dogs (SL161, SL163 and SL164) were infected with *Ancylostoma caninum* (100% identity to AJ920347). Dog SL161 also had worms with 99-100% identity to GenBank entry MZ681936, annotated as *Ancylostoma* spp. The fourth dog sample (SL165) was contaminated with nematodes presumably of the genus *Caenorhabditis* (98% identical with MH590240, *Caenorhabditis briggsae*), which are free-living nematodes.

As outlined in the introduction, we expected the prevalence of STHs in general and of *S. stercoralis* to be rather high in the chosen study area. The fairly high return rate of the samples in this study of 62.5% represents a good representative sample of the community, and it indicates a high degree of awareness and interest in engaging in such studies within the community. Although the Baermann after culture is considered the most sensitive of the microscopy dependent diagnostic methods for *Strongyloides* spp., its sensitivity has been estimated to be only a little more than 30% (Gelaye et al., 2021). It is therefore well possible that, in particular light, infections were missed. While participants reported no recent history of anthelmintic treatment, this possibility cannot be definitely excluded. In this context, it is noteworthy that not only no *Strongyloides* but also no hookworms were detected, although the culture method used was suitable for the detection of these parasites. We do believe that the non-detection of *S. stercoralis* in our samples was despite proper handling and analysis of the samples. All samples from Nuwara Eliya (human and dog) were treated the same. Four out of five dog samples contained nematodes, indicating that culture and diagnostic procedures, in particular the transportation, were appropriate. Also, the fact that three human samples contained free-living nematodes indicates that the conditions were suitable for nematode survival. We therefore believe that our result reflects a low worm burden, but not necessarily a complete absence of *S. stercoralis* in Nuwara Eliya at the time of sampling. Recently, there have been continued and increasing efforts to elevate living standards in the plantation sector along with education in the community. Our study may be an indication that these measures were effective, at least in this particular tea estate community. From Anuradhapura, the sample size was too low to come to any conclusion. However, the absence of detection should be interpreted with caution, and continued monitoring using methods optimized for sensitivity is warranted. This study was designed to isolate *S. stercoralis* individuals for molecular genotyping in order to extend the geographic range from which *S. stercoralis* molecular genetic/genomic information is available, and it was not optimised to assess the prevalence of *S. stercoralis*. Therefore, only diagnostic techniques that allow the isolation of live individual worms (i.e. Baermann funnels) were used. Given the limited number of surveys and the very different estimates of *S. stercoralis* prevalence in Sri Lanka (Buonfrate et al., 2020; Jayakody et al., 2024), large-scale epidemiological studies utilising multiple diagnostic methods would be very desirable, preferably in the context of more general STH prevalence studies.

### 3.2 Animal stool samples from the premises of the Rajarata University of Sri Lanka

Based on morphological and molecular taxonomic (HVR-I and/or *cox*-1) analyses, we found 16 out of 29 monkey samples to be positive for *Strongyloides* and for 15 of them the molecular analysis strongly suggested that these worms belonged to the species *S. fuelleborni*. In the remaining sample, the number of worms was very low and the molecular analysis failed.

**Figure 1:**
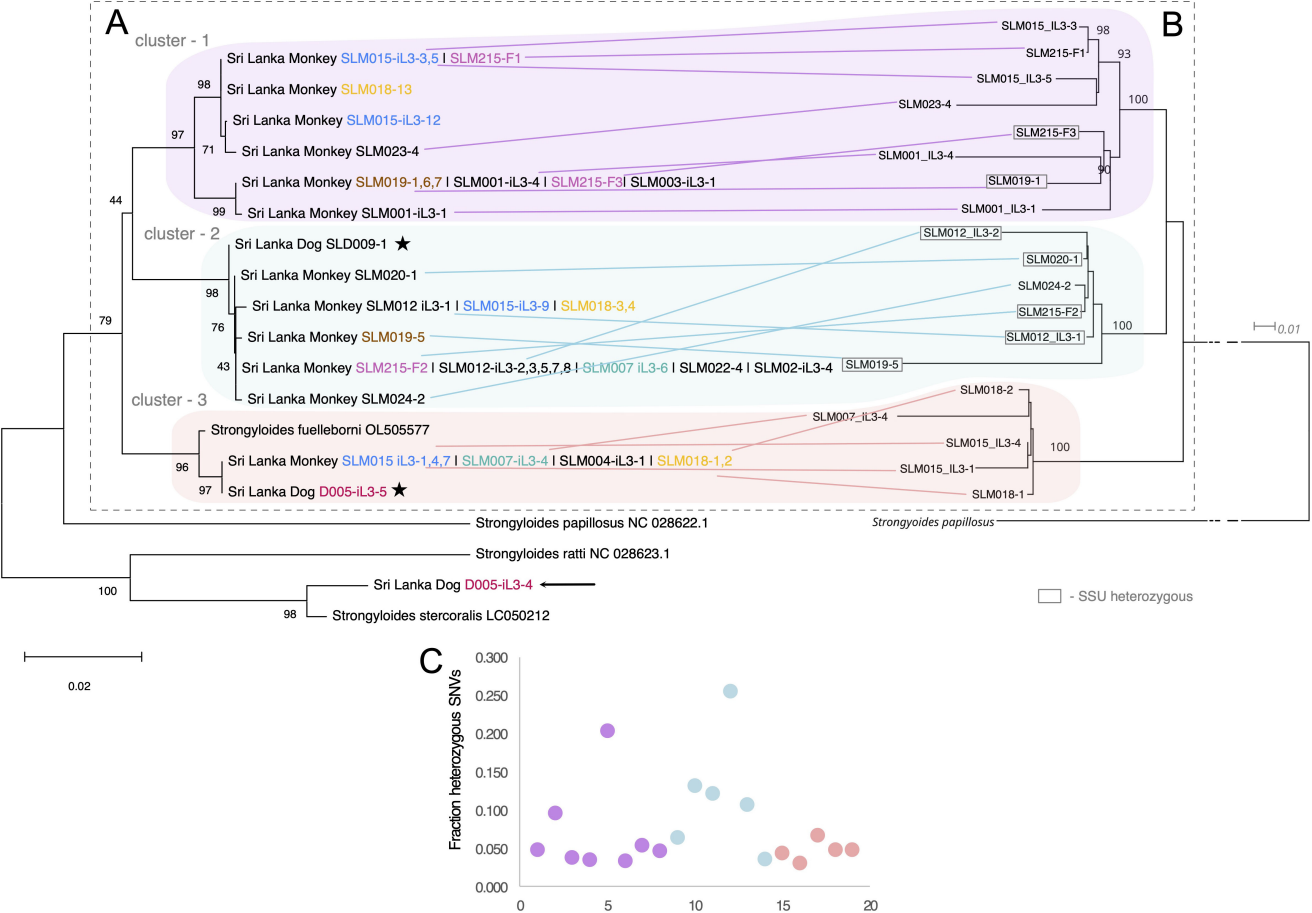
Phylogenetic relationship of the *Strongyloides* spp. isolated in this study. **(A)** Neighbour Joining tree of partial *cox-1* sequences (552bp). All *Strongyloides* spp. *cox-1* sequences found in this study are included along with selected sequences from the databases for species identification. Boot strap values based on 1000 repetitions are given. The only sequence found in monkeys and dogs is included twice (in cluster 3). To our knowledge, no *Strongyloides* spp. *cox-1* sequences from Sri Lanka have been reported before. The worm identifiers are composed as follows: [Host identifier consisting of three letters and a number Number] - [stage (if known) iL3=infective larva, F= free-living female, nothing=sex not known]-[worm number (if there are multiple worms from the same host, the numbers are separated by),]. Worm identifiers written in colours other than black indicate host individuals in which worms from more than one clade were found, worms in the same colour are from the same host individual. Asterisks represent the *S. fuelleborni* found in dog samples. The only *S. stercoralis* worm found in this study is indicated by an arrow. For the published sequences, GenBank accession numbers are given. For the accession numbers of the sequences from this study see **Supplementary Table 1**. Notice that the clusters 1 and 2 appear Sri Lanka specific (c.f. **Figure 2**; **Supplementary Figure 1**). **(B)** Neighbour Joining tree based on whole genome sequencing data. The labels follow the same scheme as in **(A)** Worms included in A and B are connected by lines (purple for cluster 1, blue for cluster 2 and red for cluster 3). Worms that showed a heterozygous SSU (based on the HVR-I and HVR-IV haplotypes) are boxed. Homozygous worms were homozygous for haplotypes XIV (HVR-I) and S (HVR-IV), heterozygous worms were heterozygous for haplotypes XIV and XII (HVR-I) and S and U (HVR-IV). For worms SLM001_iL3-4, SLM_019–1 and SLM_019–5 the coverage of HVR-IV was low such that the homozygous/heterozygous state could not be unambiguously determined at this locus. The three clusters of *S. fuelleborni* are shaded. Since only SNVs that were covered and homozygous in all worms and some worms showed very high incidence of heterozygous SNVs (see C), this tree is based on only 200 variable sites. **(C)** Proportion of heterozygous SNVs. Each dot represents one worm. The worms are ordered from 1 to 19 as in B from top to bottom (1 is SLM15_IL3_3, 19 is SLM18_1). The colours indicate the cluster.

In 3 out of 8 dog samples, we found nematodes in small numbers. Both *cox-1* sequences from sample SLD010 were 90% identical with GenBank entry LC372833.1, which is from *Gurltia* spp. Both other dog samples (samples SLD005 and SLD009) were positive for *S. fuelleborni* based on HVR-I sequences (100% identical with GenBank entry AB677955.1) and *cox-1* sequences (see **Figure 1**). One (sample SLD005) was also positive for *Ancylostoma* spp. (99% identity to the GenBank entry MZ681936.1), and one worm with a 98% identity to the GenBank entry OM976832.1 (*Panagrolaimus* spp.). Based on the *cox*-1 sequence, one of two sequenced *Strongyloides* spp. of dog SLD005 was *S. stercoralis*, while the other one was *S. fuelleborni* (see **Figure 1**).

In total, *cox*-1 sequences from 39 individual worms, representing 14 monkey samples and two dog samples, were used to generate the NJ tree shown in **Figure 1**. Identical sequences were included in the tree only once, unless they were derived from different host species. There were 12 different *cox-*1 sequences identified in monkeys and two different sequences in dogs, of which one sequence was found in both host species. The *S. fuelleborni* samples from Sri Lanka clustered with the reference *S. fuelleborni* sequence (OL505577) as expected. These *S. fuelleborni* samples fell into three well-supported (bootstrap) separate clusters. The three clusters do not represent worms from the different monkey species that exist in Sri Lanka, because different worms from the same host individual can be seen in different clusters (worms written in the same colour in **Figure 1** were isolated from the same host).

**Figure 2:**
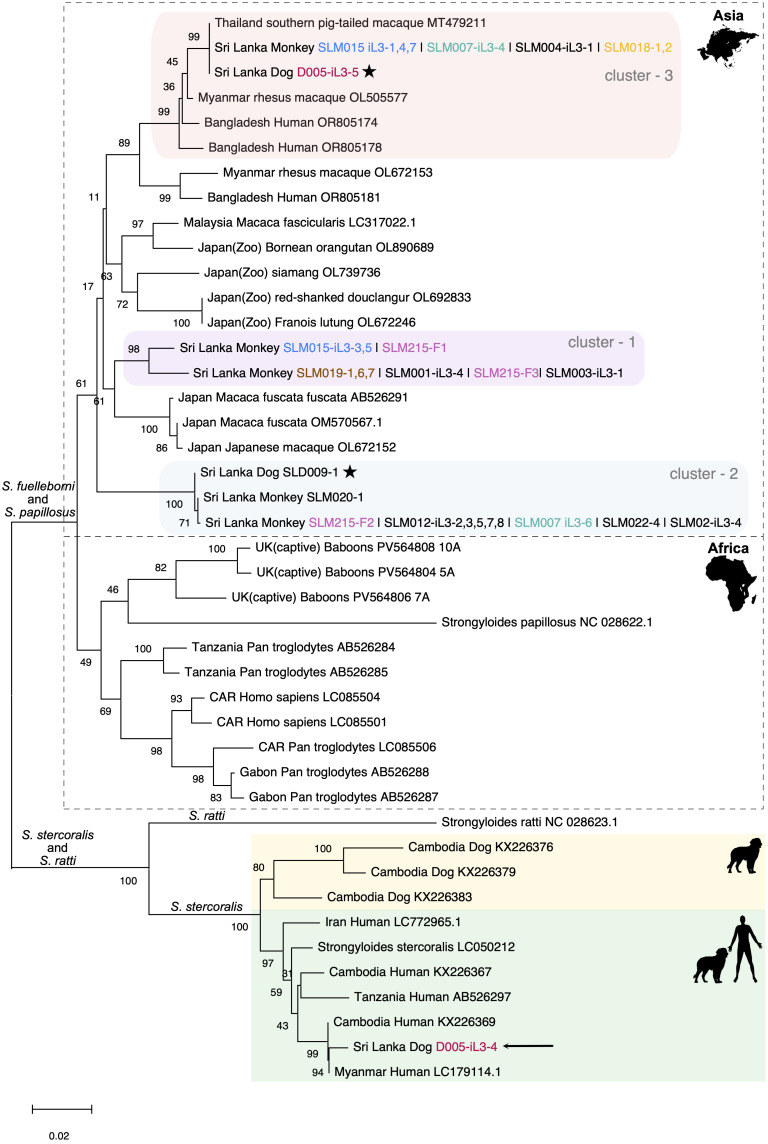
Neighbour Joining tree of selected Strongyloides *cox-1* sequences from this study and selected published *Strongyloides* sequences. Worm identifiers written in the same colour are from the same host individual. The colour scheme is the same as in **Figure 1**. Asterisks represent the *S. fuelleborni* found in dog samples. The only *S. stercoralis* worm found in Sri Lanka is indicated by an arrow. For *S. stercoralis*, the “dog only” and the “human and dog” clades (c.f. Jaleta et al., 2017; Nagayasu et al., 2017; de Ree et al., 2024) are marked in yellow and green, respectively. For the published sequences, the country of origin (CAR, Central African Republic, UK, United Kingdom), the host and the GenBank accession numbers are given. For an extended version of the figure with all the samples from this study and more published data see **Supplementary Figure 1**.

In order to see where the Sri Lankan samples fall within the global phylogeny of *S. fuelleborni* and *S. stercoralis*, selected *S. fuelleborni* and the one *S. stercoralis* sequence from Sri Lanka were used to generate a NJ tree with selected published sequences (**Figure 2**; for an extended version of the figure with all the samples from Sri Lanka and more published data see **Supplementary Figure 1**). It was made sure that, for each Sri Lankan sequence, the best non-Sri Lankan BLAST hit was included. The representative sequence from cluster 3 of **Figure 1** clusters clearly with some sequences from Asia and is 100% identical with MT479211.1, which originates from Thailand. The representative sequences from clusters 1 and 2 also fall within the previously proposed (Barratt and Sapp, 2020; Richins et al., 2025) Asian clade of *S. fuelleborni* (although not with very high bootstrap support), but they do not group with high support with any of the published sequences. Therefore, these two clusters, for the moment, appear Sri Lanka-specific. The one *S. stercoralis* sequence we found from a dog in Sri Lanka falls within the previously proposed (Jaleta et al., 2017; Nagayasu et al., 2017) human and dog shared cluster, very close to a sequence previously found in Myanmar (**Figure 2**).

For 19 of the monkey-derived worms, representing all three *cox-1* clusters, we performed single-worm short-read WGS. From these data, we extracted the *SSU* HVR-I and HVR-IV sequences. 12 worms had the HVR-I haplotype XIV and 7 were heterozygous for the haplotypes XIV and XII [**Supplementary Table 1**, for *SSU* HVR haplotype nomenclature see Richins et al. (2025)]. For three worms (SLM001_iL3-4, SLM_019–1 and SLM_019-5) the coverage of HVR-IV was very low such that the homozygous/heterozygous state could not be unambiguously determined. In all clear cases, HVR-I homozygous worms were homozygous for haplotype S at HVR-IV and HVR-I heterozygous worms were heterozygous for the haplotypes S and U at HVR-IV. While both, *SSU* homozygous and *SSU* heterozygous worms, were isolated from multiple monkeys, only in one sample (SLM215), both types were observed within the same host individual. From the WGS data we reconstructed a genome wide NJ tree (**Figure 1B**) using the *S. papillosus* genome as reference, for lack of an available *S. fuelleborni* reference genome (see materials and methods). This tree showed the same three clusters as the *cox-1* tree. We noticed that the different samples showed very variable proportions of SNVs in heterozygous state (**Figure 1C**). This might be indicative for a rather recent influx of a new genotype. The reproductive system with only occasional sexual reproduction (reviewed in Streit, 2017, ) and the fact that *S. fuelleborni* has only two chromosomes (Hunt et al., 2016) may cause large genomic regions to remain not broken by recombination (leading to a strong linkage disequilibrium) for extended periods of time after a hybridisation event. These regions are then as a whole (at all variable positions introduced by the hybridisation even) either homozygous or heterozygous, leading to lower or higher heterozygosity. It is interesting to note that the HVR-I haplotype XII and the HVR-IV haplotype U had so far only been found in Africa, while the HVR-I haplotype XIV and the HVR-IV haplotype S had only been seen in Asia (Barratt and Sapp, 2020; Richins et al., 2025). To our knowledge, this is the first observation of *S. fuelleborni* that appear to be hybrids between the Asian and the African clades. However, this conclusion that is based only on the ribosomal locus, must be taken with care and should be confirmed by comparing the alleles in the highly heterozygous worms with whole genome data from Asia and Africa, the latter of which is currently not available.

We noticed that extracting the *SSU* HVR sequences and the *cox-1* sequences from whole genome sequencing was not always as straight forward as one would expect for multi copy loci. Specifically in some samples the read coverage dropped dramatically in the HVRs or parts of the *cox-1* locus, such that from some worms some sequences could not be extracted. One possible reason might be that in these samples there was a sequence present (either homozygous or heterozygous) that was just too different from the reference sequences used (AB272235.1 for the *SSU* and OL505577 for *cox-1*) for the reads to align. This may also have led to failure of detecting additional divergent haplotypes. Therefore, high-quality *de novo* genome assemblies for *S. fuelleborni* from different geographic locations would be highly desirable.

Although patent experimental infections of dogs with *S. fuelleborni* have been reported to be possible (Sandground, 1925), to our knowledge, this is the first report of naturally occurring *S. fuelleborni* in dog samples. Currently, we cannot tell if these dogs were really fully infected with S*. fuelleborni*. Our findings might represent a transient infection or even passing through of larvae upon coprophagy. However, even if the dogs were not solidly infected with *S. fuelleborni*, they still may contribute to the soil contamination and transmission of *S. fuelleborni*. This observation, therefore, warrants further investigation into considering dogs as possible alternative hosts for *S. fuelleborni*.

It should also be highlighted that many of the studies from Sri Lanka mentioned above reported *Strongyloides* prevalence in dogs and monkeys based on egg counts. One such study (Perera et al., 2013) was also considered for the global *S. stercoralis* prevalence prediction studies for dogs (Eslahi et al., 2022). However, since *S. fuelleborni* sheds eggs, while already hatched larvae are passed in the faeces in *S. stercoralis* (Ashford, 1989; Viney et al., 1991), the authors of these reports may in fact have observed *S. fuelleborni* in dogs but not recognized them as such.

It is important to notice that our monkey and dog samples were collected from the University premises, which also include student hostels. The university premises are surrounded by forest and rich in greenery with ample amounts of large fruit trees that attract monkey troops. There are also free-roaming dogs within the university premises. In this large setting, both monkey and dog faeces are not actively cleared from public areas such as paths, the university pool and the immediate vicinity of hostels where students may come into frequent contact with these faeces. Given that we found possibly human infective *S. stercoralis* in a dog and the recent increase in reports of *S. fuelleborni* infections in humans in Asia, it might be necessary to check the students for infection with *S. fuelleborni*, especially since some of the Sri Lankan *S. fuelleborni* samples clustered together with the *S. fuelleborni* samples found in humans in Bangladesh (**Figure 2**; **Supplementary Figure 1**).

## 4 Conclusions and implications

The failure to detect *S. stercoralis* in a community in the plantation sector should not be interpreted as a complete absence of these parasites in this community but is more likely to reflect a low infection burden, suggesting that long-term improvements in living conditions and access to treatment may have reduced transmission. However, continued surveillance remains necessary.

The presence of *S. stercoralis* and *S. fuelleborni* in the faeces of free roaming monkeys and dogs within the University premises, including the dormitory and the recreational areas, i.e. the swimming pool area, raises concerns of possible exposure of the University community to these parasites despite sanitary conditions that render human to human transmission unlikely. The presence of possible hybrids between the African and the Asian clade of *S. fuelleborni* is interesting but requires confirmation that is based on additional, preferably entire genome sequence information. To this end additional sequence information from Africa is required. Our findings suggest that dogs are also a factor in the contamination of soil with *S. fuelleborni*. It remains to be elucidated if the *S. fuelleborni* shed by dogs are really the result of true infections or rather due to coprophagy.

## Data Availability

The datasets presented in this study can be found in online repositories. The names of the repository/repositories and accession number(s) can be found in the article/**Supplementary Material.**

## Supplementary material

Supplementary Figure 1 Neighbour Joining tree with all Strongyloides cox-1 sequences determined in this study and selected published (S) fuelleborni sequences. Worm identifiers written in the same colour are from the same host individual. The colour scheme is the same as in **Figure 1**. Asterisks represent the (S) fuelleborni found in dog samples. The only (S) stercoralis worm found in this study is indicated by an arrow and serves as an outgroup. For the published sequences, the country of origin (CAR, Central African Republic, UK, United Kingdom), the host and the GenBank accession numbers are given.
