# Supplementary figures and images for: Molecular taxonomic study of Strongyloides spp. in Sri Lanka with emphasis on Strongyloides fuelleborni on the premises of a Sri Lankan university
Source: Front Parasitol. 2026 Jul 8;5:1821438. doi: 10.3389/fpara.2026.1821438 (PMC13388871; doi:10.3389/fpara.2026.1821438)

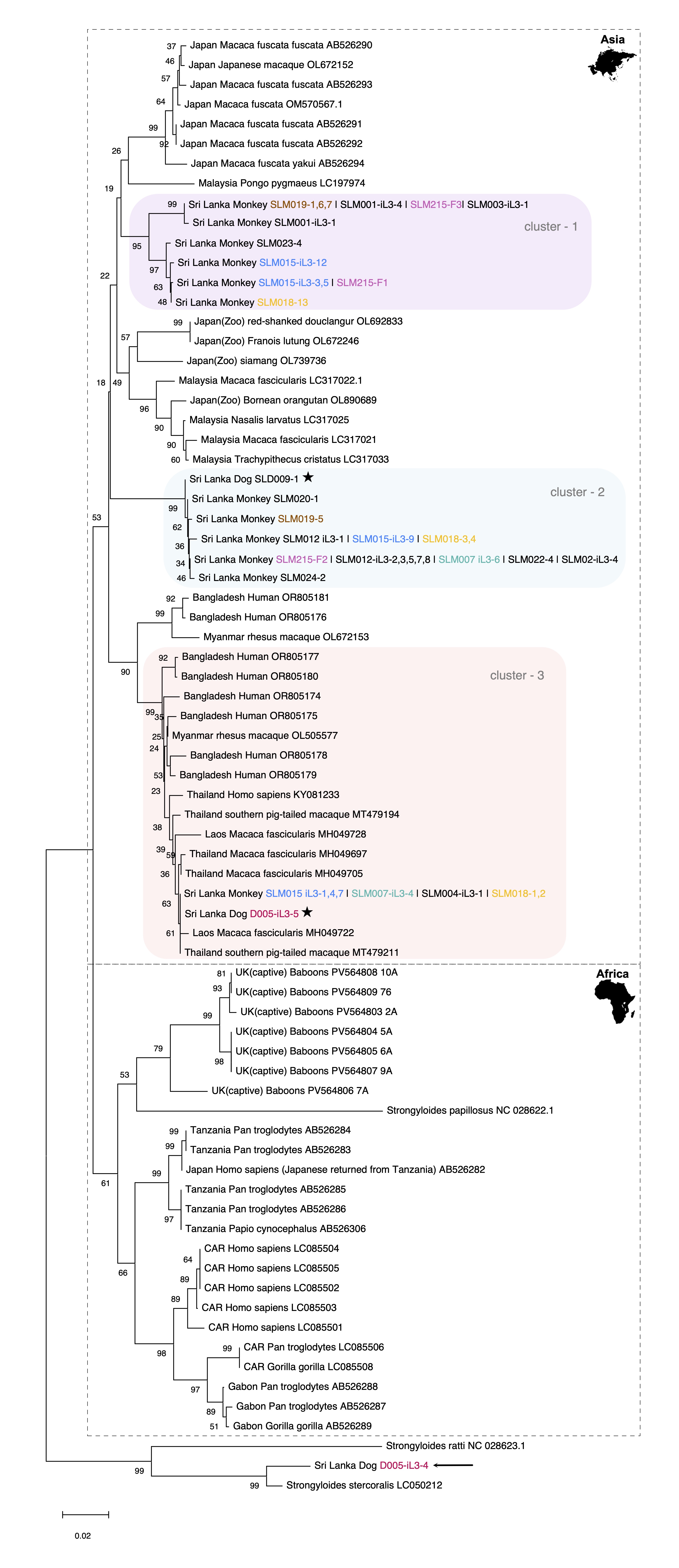

Supplement: Supplementary Figure 1 — Neighbour Joining tree with all Strongyloides cox-1 sequences determined in this study and selected published (S) fuelleborni sequences. Worm identifiers written in the same colour are from the same host individual. The colour scheme is the same as in Figure 1. Asterisks represent the (S) fuelleborni found in dog samples. The only (S) stercoralis worm found in this study is indicated by an arrow and serves as an outgroup. For the published sequences, the country of origin (CAR, Central African Republic, UK, United Kingdom), the host and the GenBank accession numbers are given. [file Image1.jpeg]
